# Analysis, Design and Realization of a Furnace for In Situ Wettability Experiments at High Temperatures under X-ray Microtomography

**DOI:** 10.3390/jimaging7110240

**Published:** 2021-11-15

**Authors:** Roberto Fedele, Fareeha Hameed, Nicola Cefis, Gabriele Vergani

**Affiliations:** 1 Department of Civil and Environmental Engineering (DICA), Politecnico di Milano, 20133 Milan, Italy; fareehahameed@fccollege.edu.pk (F.H.); nicola.cefis@polimi.it (N.C.); gabriele1.vergani@mail.polimi.it (G.V.); 2 Elettra-Sincrotrone Trieste S.C.p.A., 34149 Trieste, Italy; 3 The ‘Abdus Salam’ International Centre for Theoretical Physics, 34151 Trieste, Italy; 4 Department of Physics, Forman Christian College (A Chartered University), Lahore 54600, Pakistan

**Keywords:** X-ray microtomography, in situ experiments, wettability, brazing joints, materials science

## Abstract

In this study, we analyzed the problem of a compact furnace, to be used for in situ experiments in a cone-beam X-ray microtomography commercial system. The design process was accomplished and outlined through its main steps, until the realization of a prototype. The furnace was conceived to carry out wettability experiments at temperatures up to 700 °C and under inert atmosphere on sessile droplets of a molten metal alloy, with a few millimeters diameter, posed on a thin ceramic substrate. X-ray imaging of the molten droplet is expected to permit an accurate three-dimensional reconstruction of the droplet profile and a robust estimation of the related quantities (such as the contact angle and the surface tension) utilized for the assessment of metal-ceramic joints by brazing. The challenges faced during this project, mostly related to the constraints of the setup, and the novel solutions implemented were discussed also with the support of analytical and numerical tools, in terms of interaction of X-rays with matter, geometry and working principle, heat transfer and insulation, material selection.

## 1. Introduction

Fully three-dimensional, high accuracy imaging by X-ray computed microtomography (see e.g., [1]) represents an extraordinary and flexible tool to improve the conventional experiments on materials, fostering research and innovation. As is well known, the X-ray beams, generated from a synchrotron source or by a laboratory tube, can penetrate an opaque sample; the attenuated images, revealed by a scintillator and digitized by a camera, through the tomographic inversion, provide information on the bulk material, to be interpreted in a qualitative or a quantitative fashion; see [2,3]. In situ experiments, namely, tests monitored in real time by an X-ray system, represent well-established, modern techniques in diverse disciplines, favored by the improved spatio-temporal resolution (down to submicron and millisecond scales), although the big amount of data often requires off-line processing [4]. Among the numerous contributions available in the recent literature, we can mention the following: Hameed et al. [5], investigating the damage mechanisms in SiC_f_/SiC samples under in situ compression loading at room temperature; Patterson et al. [6], who studied the mechanical response of hyperelastic polymer foams by a loading apparatus utilizing a polymethyl methacrylate (PMMA) shell to reduce the attenuation; the research [7] by Zambrano et al., where the flow of the deionized water in grainstone samples was monitored through 2D dynamic radiography and 3D tomography by a spallation neutron source, with bimodal imaging (X-rays and neutrons, sequentially); the study [8] by Tötzke et al., who made recourse to an ultrafast neutron tomography to track the dynamic water flows through a porous soil column. In this respect, several technological features of the tomographic systems play a crucial role, such as the mechanical and thermal stability of the setup during the acquisition process or the effective coupling with the auxiliary apparatus.

Often, advanced applications require furnaces with an innovative design for ex-situ heating. For instance, Tosti et al. [9] developed a high temperature oven where a SiC sample could rotate into a lithium-lead bath, to assess the erosion and the corrosion of the composite; Lee et al. [10] realized a vacuum furnace for a fluxless bonding process, inhibiting the oxidation of the solder whilst it is being melted. In the context of the X-ray microtomography by a synchrotron light, Bellet et al. [11] realized a water cooled furnace equipped with a quartz glass chamber, to carry out in situ experiments and study the microstructural evolution of material samples; Grupp et al. [12] developed an in situ radiation furnace utilizing halogen heating lamps, water and air cooled; Haboub et al. [13,14] realized a large-size and presumably heavy assembly, mounted on a portable rotary stage, coupling an oven under vacuum for ultrahigh temperatures with a loading apparatus for tension/compression, both water cooled for thermal stability. It is worth emphasizing that the synchrotron beamlines can usually accommodate large size equipment for in situ testing, which exploit also the superior quality of the light for different features.

The above perspectives gave rise to the attempt of monitoring through X-ray microtomography the wettability experiments of a liquid droplet on a solid substrate, referred to as sessile drop tests. At room temperature under air, Santini et al. [15] investigated drops gently deposited on a substrate. At high temperatures, such tests must be carried out under inert gas or under high vacuum, to prevent the oxidation that significantly modifies the surface features of the droplet and, with them, the wetting behavior. These experiments are widely utilized in materials science for the assessment of the metal-ceramic brazing joints through measurements of the contact angle, see e.g., [16,17,18,19]. The ultimate aim is to supersede the conventional approach, based on a 2D side view of the droplet (see [20]), with an accurate three-dimensional reconstruction of the droplet profile at rest provided by the X-ray microtomography [15].

This paper deals with the design, the analysis and the realization of a novel experimental equipment, that allows the sessile drop experiments at high temperatures (700 °C) and under inert atmosphere to be performed in situ through a laboratory X-ray setup.

## 2. Materials and Methods

**Figure 1:**
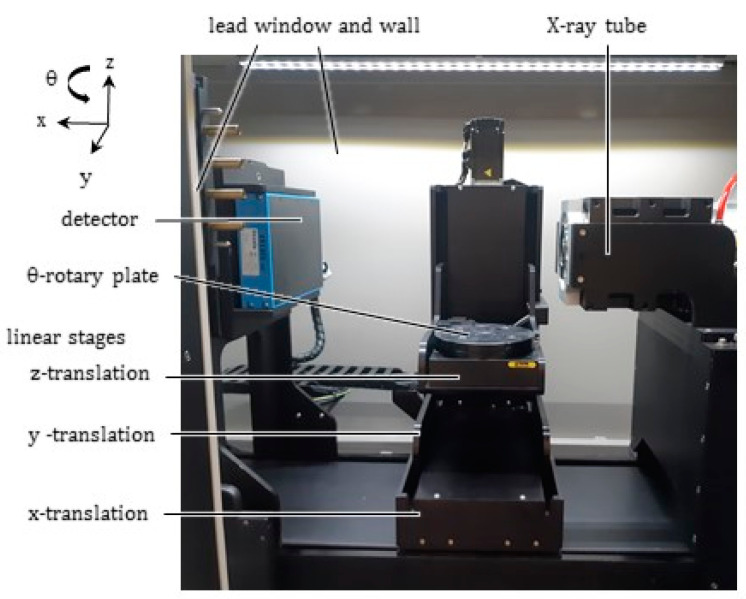
Inner view of the cabinet for X-ray microtomography available at Politecnico di Milano, showing the 4-dof motorized system for the sample positioning (see the reference frame), the tube and the movable detector.

The commercial system for X-ray microtomography currently available in the laboratory “AMALA” of Politecnico di Milano (Milan, Italy) is the X-25 model produced by NSI (North Star Imaging, US). It includes the following: an X-ray microfocus transmission tube (XRayWorkX, model XWT-160-T), with maximum voltage 160 kV, maximum current 3 mA, maximum power emission/target 80/10 W, focal spot < 2 μm; a CMOS flat panel detector (Dexela, model 1512) with a Gadox scintillator, with 145 mm × 115 mm active image area, 1944 × 1536 pixels and 74.8 μm pitch, 16 bit digitization. The tube, with a target made of beryllium as substrate and tungsten as active layer, generates a polychromatic X-ray beam by bremsstrahlung radiation, see e.g., [21]: as well known, its emitted continuum spectrum exhibits a photon energy at peak equaling the tube voltage (keVp). Figure 1 shows the inner part of the cabinet. The system with a cone beam geometry [22], apt to position independently the rotary stage and the detector along a length of almost 900 mm, is capable of geometric magnifications up to about 65× for the smallest samples, which can be shifted very close to the tube.

Which constraints the novel furnace is supposed to satisfy?

(i) As a basic constraint, the prototype must fit the limited space available within the tomographic cabinet (see Figure 1). This constraint has important consequences well beyond the geometry of the equipment, and gives rise to serious difficulties. First of all, the thickness of the insulation layers for the furnace must remain small; at the same time, the temperatures over the outer wall of the equipment have to be kept low, to avoid damaging the other components of the tomograph.

(ii) The furnace must not exceed the weight capacity of the rotary stage (herein 11 kg): surpassing that threshold, the precision of the angular rotation is no longer guaranteed.

(iii) Since the voltage and the current of the X-ray tube are not high, the design of the furnace with the selection of the constituent materials and the sealing solutions must imply as much as possible a reduced attenuation for the X-ray beam.

(iv) In a commercial, turnkey tomographic system the acquisition process is not programmable; in the simplest scenario, the sample follows the 360° rotation of the rotary plate, controlled by the built-in software. Moreover, since we intend to scan a molten droplet at rest over a substrate, it is extremely important that the rotation occurs in a stable way, with small vibrations and wobbling.

(v) As for the heating process, the furnace must attain temperatures of at least 700 °C, uniformly in the neighborhood of the sample. The materials selected for the different components must be compatible with such service temperatures; their thermal expansion must be taken into account for the design solutions.

**Figure 2:**
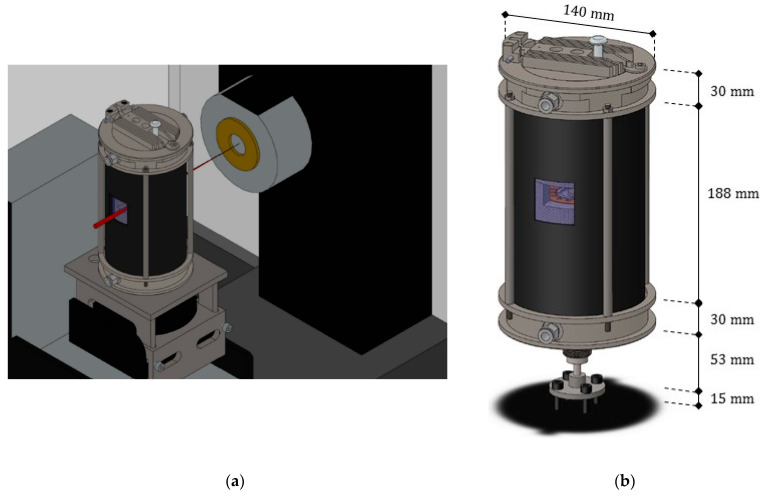
(**a**) Axonometric view of the furnace positioned within the cabinet, with a pictorial representation of the X-ray beam (colored in red) emitted by the tube; (**b**) enlarged view of the prototype, with overall dimensions.

On the basis of the general considerations (i)–(v), it appears clear that several problems were implied in the development of the novel equipment. Figure 2 shows a drawing of the prototype within the tomographic setup, with some dimensions. A list of the main components of the prototype was included in Table A1 (Appendix A), which specifies their role and the materials selected together with a reference code, recalled in the text and in the figures for better clarity.

Before going into details, it is worth emphasizing a design choice we assumed at the beginning of this study and affected all the subsequent steps. Inside the furnace, exclusively the sample holder, on which the droplet/substrate pair rests, can rotate fastened to the rotary stage of the tomograph through a properly insulated transmission system. The heating element (with all its cables and wires) as well as the multilayer structure surrounding the test chamber remain still during the whole scanning process, clamped to the translatory stage of the tomograph. By this choice we solved two problems at once: we prevented complications due to rotating twisted wires, stiffened by the insulation coating; secondly, we permitted an accurate positioning of the entire furnace with respect to the X-ray source, and a full control of the geometric magnification within the cone beam geometry.

**Figure 3:**
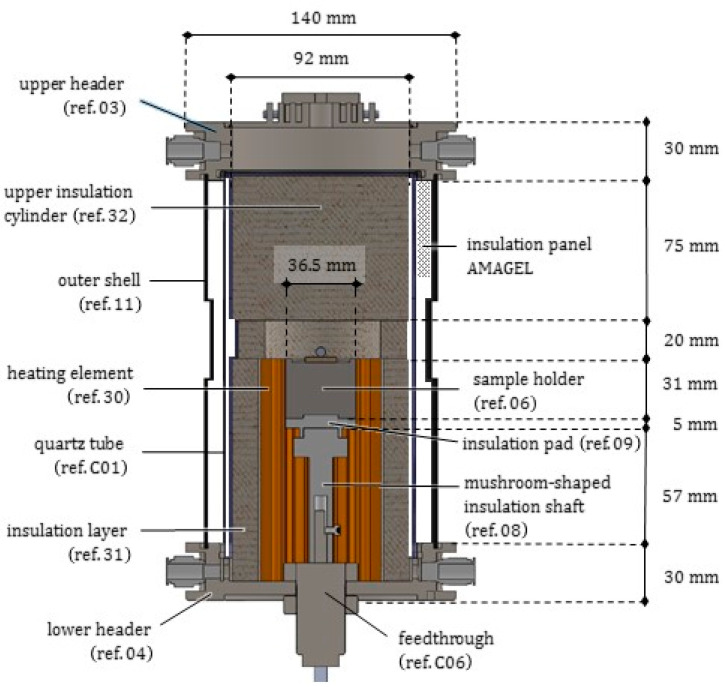
Longitudinal radial section of the furnace prototype. Main components and dimensions are indicated.

**Figure 4:**
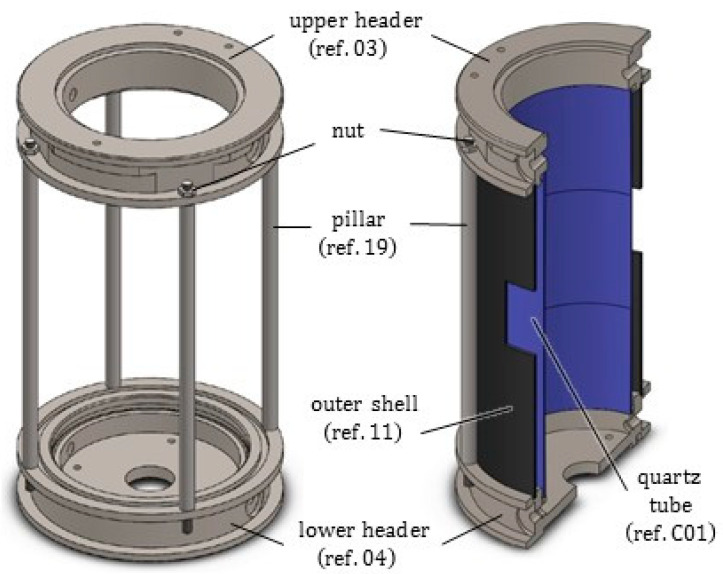
Upper and lower header of the furnace, fastened by four pillars (ref. 19). The outer metal shell (marked by black color) and the quartz glass tube (in blue) rest inside the grooves of the two headers. In both the headers, the lateral ducts for the inert gas can be recognized.

As depicted in Figure 2 and Figure 3, the novel equipment possesses a cylindrical geometry, with outer diameter 144 mm and height 250 mm, including within its interior several coaxial layers. The outer surface is constituted of a metal shell (ref. 11) with some openings, playing at the same time a structural and a protection role, closed at the top and at the bottom by two cylindrical steel headers (ref. 03 and ref. 04). Such headers are connected to each other by four pillars (ref. 19) fixed by screws, and, together with the outer shell, constitute a stiff skeleton for the entire apparatus, see Figure 4. Moreover, both the headers of the furnace include two lateral conduits with connectors for the gas (ref. C10). A quartz glass tube (ref. C01), sealed through O-rings (ref. C07), contains the inner atmosphere while permitting X-ray irradiation with a low attenuation.

**Figure 5:**
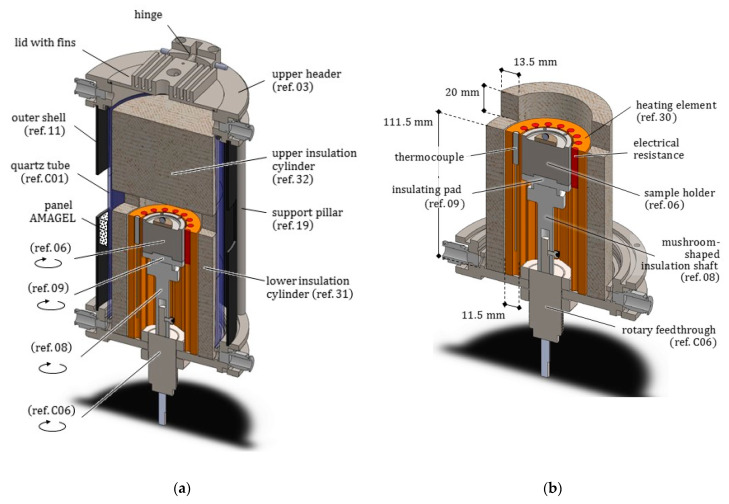
(**a**) Axonometric view of the entire furnace, longitudinally sectioned, with all the coaxial layers. The circular arrows specify the components rotating during the acquisition step. (**b**) Detail of the heating element (ref. 30) surrounding the sample holder (ref. 06) with its rotation transmission system, both enclosed by the insulation layer with the two openings for the X-rays beam. In both the headers, the two lateral connections for the inert gas pipes appear sectioned.

**Figure 6:**
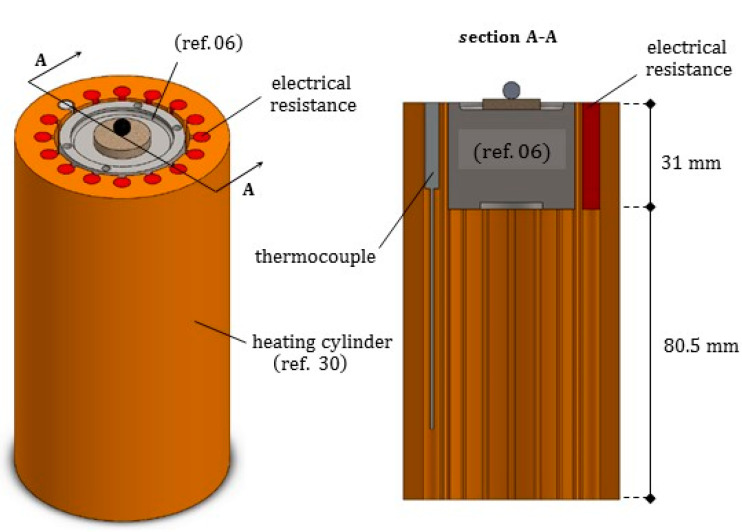
Axonometric view and longitudinal radial section of the heating ceramic element, including in its vertical grooves the electrical resistances (colored in red) and the thermocouple (in grey), and surrounding the sample holder.

As represented in Figure 5, in the core of the furnace a compact metal cylinder (ref. 06) serves as a sample holder for the molten droplet/ceramic substrate pair. It is fastened to special components transmitting the rotation and providing an adequate insulation to the lower parts of the furnace. The sample holder is surrounded by the heating element (ref. 30), see Figure 6. The heating element in turn is enclosed by a cylindrical insulation layer (ref. 31), extending from the base up to 20 mm beyond its top surface (aligned with the base of the sample). Two small rectangular openings in the insulation layer were positioned along the path of X-ray beam, to reduce as much as possible its attenuation.

Consistently, the outer metal shell (ref. 11) also includes two openings at the corresponding points. The compact roof of the furnace chamber (ref. 32) exhibits a cylindrical shape which prolongs vertically the outer insulation layer (ref. 31) up to the furnace lid.

The entire furnace is anchored to the translation stage of the tomograph by means of a massive metal support, easily removable. Such a support was conceived to reduce vibrations as much as possible and to permit an accurate positioning of the furnace without modifying the preexisting setup.

After a general outline of the main features of the furnace prototype, we now provide a description of the single components, highlighting the design choices and the materials selected.

### 2.1. Quartz Glass Tube

The inner part of the furnace is enclosed by a 2 mm thick quartz glass tube (ref.C01), with inner radius 48 mm and height 200 mm; it exerts a confinement for the inert gas volume filling the test chamber and the connected cavity, see Figure 3, Figure 4 and Figure 5. The tube, made of a high purity fused silica, was provided by Squall International bv (NL). Quartz glass exhibits the following main features: (i) a low X-ray attenuation; (ii) a maximum operating temperature of 1100 °C (with a peak temperature of 1300 °C) and a good thermal shock resistance; (iii) low thermal expansion. Some of its properties are listed in Table A2. As a drawback, quartz is a brittle material, and the limited workability implies tolerances higher than metals: the geometry of the tube transversal section cannot be guaranteed with a high accuracy (in practice, the cross section of the tube turns out to be slightly elliptical) and may vary along the height. The ends of the quartz tube are housed inside the furnace headers and sealed by means of O-rings, able to resist up to 350 °C, which cushion elastically the possibly irregular profile and its thermal deformation in the horizontal plane; along the vertical direction, the upper end of the tube is free to expand in a cavity left within the upper header.

### 2.2. Furnace Headers

The cylindrical headers of the furnace (see Figure 4), one for the upper end (ref. 03) and the other one for the base (ref. 04), were made of AISI 304. AISI 304 stainless steel is less conductive than carbon steel and exhibits a high workability; some of its properties are listed in Table A3.

The headers accommodate the outer metal shell (ref. 11) into a groove of their perimeter. The lower header (ref. 04) is constituted in its interior of a compact, circular plate with a few, very small peripheral holes (each one with diameter 4 mm) for cables and wires, and a central hole with diameter 26 mm for the insertion of the rotary motion feedthrough (ref. C06). To permit the inspection and the positioning of the sample, the upper header is equipped with a hinged lid (100 mm diameter), partly finned to enhance cooling. Both the headers possess two lateral ducts, equipped with outer connections for the gas pipes, which appear sectioned in Figure 3. To favor the gas circulation, a small hole can be drilled also at the base of the insulation layer and of the ceramic crown.

### 2.3. Sample Holder

The sample holder is a compact cylinder (ref. 06) with diameter $\phi_{06}=36.5\;\mathrm{mm}$ and height $l_{06}=31.0\;\mathrm{mm}$, made of $\mathrm{HASTELLOY}\;C22^{®}$, a nickel-chromium-molybdenum alloy with a melting temperature of 1399 °C. $\mathrm{HASTELLOY}\;C22^{®}$ exhibits high ductility, excellent weldability and high resistance to both oxidizing and non-oxidizing chemicals; for these reasons, it is widely utilized in reactors and heat exchangers. Its properties were included in Table A4.

**Figure 7:**
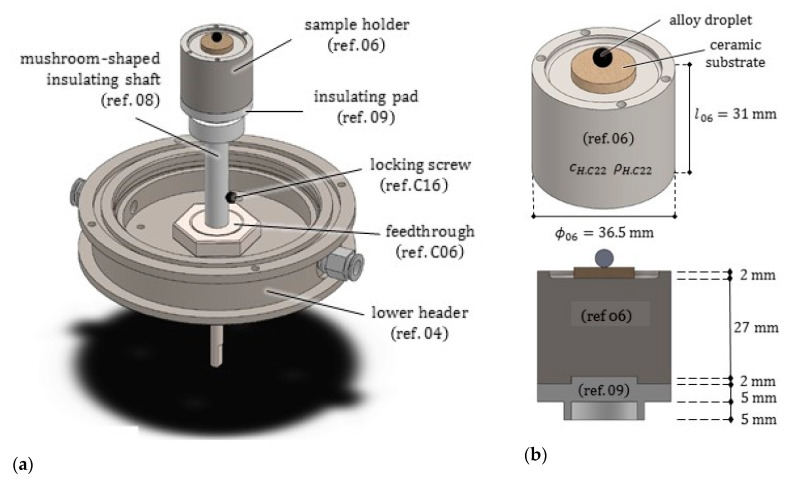
(**a**) Axonometric view of the rotating sample holder and of its transmission system. The lower header (ref. 04) of the furnace stands still during scanning. (**b**) The sample holder and its longitudinal radial section, from which the insulating pad (ref. 09) can be recognized.

The piece includes a 2 mm deep recess in the upper part, to house safely the molten droplet/ceramic substrate pair. As it can be seen in Figure 7, another cylindrical recess was realized in the lower part of the piece, to provide a secure connection with the insulating pad (ref. 09).

### 2.4. Rotation Transmission System

The rotation transmission system, coaxial with the central axis of the furnace, connects the sample holder to the rotary stage of the tomograph. It is constituted of the following components, as illustrated in Figure 5 and Figure 7: from above to below, the insulating pad (ref. 09), the insulating shaft (ref. 08), the feedthrough (ref. C06) and a screwed connection (ref. 20). Its main role is to transmit effectively the rotation, in such a way that during the radiograph acquisition, the sample rotates together with the rotary stage of the cabinet. Moreover, the rotation transmission system must provide an effective thermal insulation, since it realizes a thermal bridge between the hot sample and the rotary stage of the tomograph, and has to prevent gas leaking through the lower base.

The rotary motion feedthrough (ref. C06), provided by ANCORP (US) with the main parts of stainless steel, by its 110 mm length transmits rotations from the outside to the inside of the furnace lower header. It exhibits a maximum operating temperature of about 400 °C, since one of its parts is sealed by an elastomer. The two ball bearings in it allow a smooth and friction-free rotation of the shaft around its axis.

Since the thermal conductivity of the stainless steel is high, namely k=17 W/(m·°C), to minimize the heat transfer the feedthrough was inserted coaxially inside a cylindrical groove realized at the lower end of the mushroom-shaped shaft (ref. 08). Such an insulation component was worked by a numerical control machine from a prismatic block of $\mathrm{ORVICAL}\;1500^{®}$. The same material was utilized for a further component, referred to as insulation pad (ref. 09), positioned inside the lower recess of the sample holder. $\mathrm{ORVICAL}\;1500^{®}$ is an asbestos free compound material based on heat-treated calcium silicate with a low thermal conductivity. Its long-term operating temperature can attain 1500 °C, with peaks of 1600 °C. 

In a preliminary phase of this study the glass ceramic ${\mathrm{MACOR}}^{®}$ and the calcium silicate based ceramic $\mathrm{DURATEC}\;750^{®}$ were also considered as possible candidates. The mechanical and thermal properties of these three materials were reported in Table A5.

It is worth considering that, along with many engineering materials, the porous ceramics are subjected to outgassing, namely to the release of gas absorbed or trapped, which can contaminate the test chamber, see e.g., [23]. However, the outgassing rate, generally worsened by the high temperatures, can vary by several orders of magnitude from material to material, or also for the same sample as a consequence of chemical/physical treatments, such as the polishing/cleaning of the surfaces by suitable products, the exposure to UV rays or the “bake-out”, namely a combination of heating and vacuum cycles [24]. A commonly adopted procedure to reduce the contamination level consists of blowing inside the test chamber an inert gas with a high or ultra high purity, at a pressure slightly higher than the atmospheric pressure, acting as a sort of flushing.

### 2.5. Heating Element

The heating circular crown (ref. 30, see Figure 5 and Figure 6) has the outer and inner diameters equal to 62.4 mm and 39.3 mm, respectively. It was realized by a refractory ceramics with commercial name 42TE, produced from fireclay. The ceramic element was realized by superimposing two precast cylinders: the upper one surrounds the sample holder, with height 31 mm, has several holes housing the electrical resistances capable of providing a maximum electrical power of 1100 W, and one thermocouple; the lower one serves as a base, posed onto the inferior header. A 1.4 mm thick cavity was left between the heating cylinder and the coaxial sample holder (ref. 06), to guarantee no contact conditions during the rotation, when including the thermal deformation.

### 2.6. Thermal Insulation

The material ${\mathrm{PROMAFORM}}^{®}$ was selected to realize both the cylindrical layer (ref. 31) surrounding the heating element, and the compact cylindrical roof (ref. 32), extending from the top surface of the lateral insulation layer up to the furnace lid, see Figure 5 and Figure 6. The brand name ${\mathrm{PROMAFORM}}^{®}$ indicates a variety of high-quality insulation sheets made of refractory ceramics or of bio-soluble fibers, especially suitable for high temperatures. They exhibit a high workability and an excellent resistance to flame and to thermal shocks. In particular, ${\mathrm{PROMAFORM}}^{®}\;\mathrm{ECO}\;1250$, whose properties were included in Table A6, is certified as not carcinogenic.

To reduce the X-ray attenuation, two openings, 20 mm high, were realized within the insulation cylinder (ref. 31), at the same level of the sample. Due to the cone beam setup, the window closest to the source was created 20 mm wide, the opposite one, 30 mm. The window size was optimized to include within the field of view drops and substrates of diverse dimensions, at varying the magnification. Effectiveness of this choice was validated experimentally by radiographs on a phantom.

In the cavity between the quartz tube and outer metal shell, as indicated in Figure 3 and Figure 5, a further insulation panel was inserted, made of ${\mathrm{AMAGEL}}^{®}$ A2 650, provided by AMA composites (Italy). ${\mathrm{AMAGEL}}^{®}$ is a siliceous aerogel based composite, with an insulating matrix based on glass fibers and a high concentration of Airgel nanoporous foam. It exhibits a low thermal conductivity, namely $k_{\mathrm{AMA}}=0.019\;W/\left(m\cdot {}^{\circ}C\right)$, working temperatures up to 650 °C, superior flexibility, see Table A7. In correspondence of the windows, two cutouts were created in the AMAGEL panel.

### 2.7. Furnace Anchoring System

**Figure 8:**
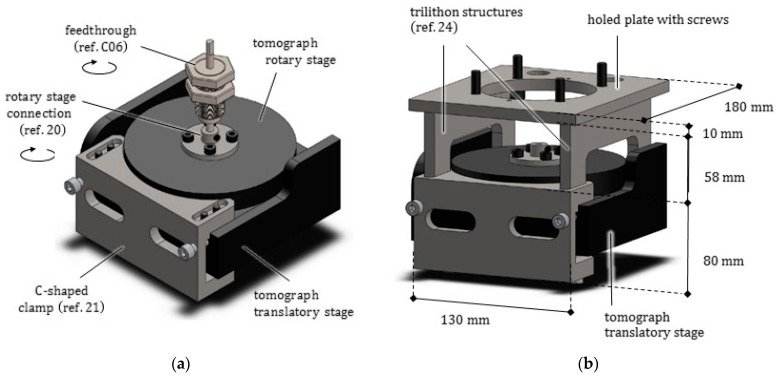
(**a**) View of the feedthrough inserted into the head of a connection component (ref. 20), in turn screwed to the rotary plate of the tomograph. (**b**) Anchoring system of the furnace, composed of C-shaped clamp (ref. 21), two lateral trilithon structures (ref. 24), and a thick holed plate permitting the passage of the connection component (ref. 20) and wires.

The support of the furnace, as shown in Figure 8, is constituted of a heavy C-clamp (ref. 21), fastened to the motorized sled of the tomograph translating along the z direction, passively advected along directions x and y (see Figure 1). Two trilithon (ref. 24) elements, screwed over the clamp, support a thick plate (ref. 24) from opposite sides. The furnace is anchored to this plate by means of screws. All these components were made of carbon steel.

## 3. Results

After a detailed description of the furnace geometry, components and materials, this Section is devoted to a quantitative assessment of basic phenomena implied by the design solutions. In particular, the attenuation of the X-ray beam was estimated experimentally by radiographs on a solid droplet at room temperature; the steady state heat transfer mechanisms were investigated by analytical and numerical approaches.

### 3.1. X-ray Interaction with the Furnace

As is well known, X-rays can interact with matter in different ways: the probability of these interactions depend on both the incident radiation and the material. As a net effect, the photons are attenuated when passing through a material layer. Assuming that the incident X-ray beam is monochromatic and that the material has a uniform density and atomic number, the attenuation can be described by an exponential relationship known as the Beer-Lambert law [22]:

(1) $$I\left(x\right)=I_{0}e^{-\mu x}$$

where symbols have the following meaning: *I*(x) and *I*_0_ denote the transmitted and incident intensities, respectively; x indicates the material thickness; *µ*(x,*E*) the linear attenuation coefficient [cm^−1^], which depends on both the energy *E* [keV] of the incident X-ray photon and the atomic number Z of the material.

To preliminarily assess the attenuation of the sample in the furnace, a irregularly hemispherical Ag solid droplet (with diameter 5.2 mm) was placed on a 1 mm thick disc of sapphire and subjected to X-ray irradiation under air, in the specific NSI cabinet. Radiographs of the sample were acquired, with and without the quartz tube. As attempt values, the tube voltage and current were tuned to 90 kV and 77 μA, respectively. The tube-to-detector and the tube-to-sample distances were set to 682 mm and 87 mm, respectively, giving rise to a 10 μm estimated voxel size. Moreover, a 0.3 mm copper filter was used.

**Figure 9:**
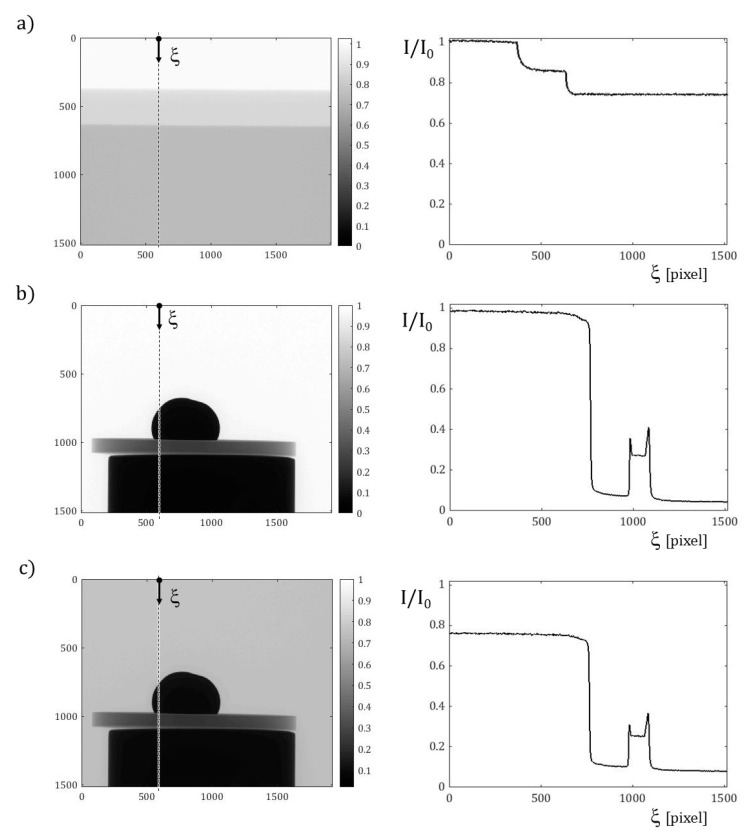
X-ray radiographs, acquired with the same beam parameters, in the left column, and relevant transmission I/I_0_ evaluated along the vertical section marked by the dashed line and run by the coordinate ξ, in the right column. Content per row: (**a**) from above to below, flat-field I_0_, 2 mm quartz and 4 mm quartz attenuated image; (**b**) Ag solid droplet on a sapphire substrate, positioned on a further support; (**c**) the same sample and angular projection, placed inside a 2 mm thick quartz tube.

These radiographs, synoptically shown in Figure 9, revealed a satisfactory contrast between the metal droplet and the ceramic substrate, whilst the transmission I/I_0_ evaluated along a vertical section confirmed that the quartz glass does not affect the results significantly. Moreover, by increasing the tube voltage to 115 kV, higher values of the transmission were found, indicating that the image quality provided by the present setup can be further improved.

Table 1 reports the values of the linear attenuation coefficient for the materials involved, at the (monochromatic) energy 50 keV, corresponding approximately to the mean value of the polychromatic spectrum generated by the present system for the experiments outlined.

### 3.2. Minimum Heat Supply

**Figure 10:**
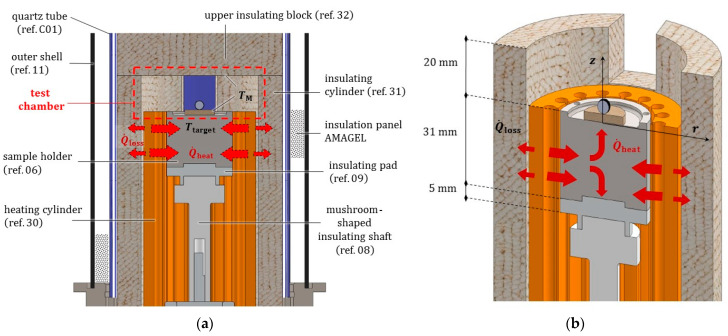
(**a**) Longitudinal radial section of the furnace deprived of the headers. (**b**) Axonometric view of the radially sectioned heating element (ref. 30), the cylindrical sample holder (ref. 06) and the insulation layer with an opening along the X-ray beam path. A pictorial representation is provided for the heat generated by the ceramic circular crown, a part of which is transferred to the sample (ref. 06) whilst another part is lost.

We can estimate the minimum amount of heat that the ceramic circular crown (ref. 30) must generate, to attain the steady state conditions in the furnace. All the phases and the components are initially at room temperature, i.e., $T_{\mathrm{start}}=30\;{}^{\circ}C$. Neglecting the energy losses through the walls of the test chamber (see Figure 10), the following contributions must be considered: (i) the heat $Q_{\mathrm{heat}}$ needed to warm up the drop to its melting temperature $T_{M}$ plus the latent heat $Q_{\mathrm{melt}}$ to complete melting; (ii) the heat $Q_{\mathrm{ar}}$ needed to warm the inert gas (argon) inside the furnace chamber up to $T_{M}$; (iii) the heat $Q_{06\;\mathrm{heat}}$ to warm the sample holder (ref. 06) to the temperature $T_{\mathrm{target}}$, with $T_{\mathrm{target}}>T_{M}$. At the point (ii) we assumed that, under steady conditions, in the test chamber the temperature of inert gas equals that of the molten drop.

(i) As a methodological reference we considered an aluminum droplet with a diameter of about 6 mm, mass *m* = 0.5 g, melting temperature $T_{M}=660\;{}^{\circ}C$, latent heat of fusion $\lambda_{M}=334\;\mathrm{kJ}/\mathrm{kg}$ and specific heat c = 869.9 J/(kg °C). By the fundamental law of thermology (see [26]), we obtain $Q_{\mathrm{drop}}=Q_{\mathrm{heat}}+Q_{\mathrm{melt}}=441\;J$.

(ii) The total volume filled with argon, say $V_{\mathrm{ar},\mathrm{tot}}$ [m^3^], includes not only the test chamber, but also two small openings in the insulation layer (ref. 31) and the outer cavity limited by the quartz tube (ref. C01). The volume of the cylindrical test chamber, with base diameter $\phi_{31,\mathrm{in}}=66\;\mathrm{mm}$ and height $h_{\mathrm{window}}=20\;\mathrm{mm}$, plus the two windows amounts to $V_{\mathrm{ar},\mathrm{ch}}=\left(\pi \;r_{31,\mathrm{in}}^{2}\right)\cdot h_{\mathrm{window}}+h_{\mathrm{window}}13.5\;\times \;\left(20+30\right)=8.1924\cdot 10^{-5}\;m^{3}$. The volume of the outer cavity, with height $h_{\mathrm{quartz}}=200\;\mathrm{mm}$, the external and internal radii equal to $r_{\mathrm{ext}}=48\;\mathrm{mm}$ and $r_{\mathrm{in}}=46\;\mathrm{mm}$ respectively, amounts to $V_{\mathrm{ar},\mathrm{ext}}=\left[\pi \cdot \left(r_{\mathrm{ext}}^{2}-r_{\mathrm{in}}^{2}\right)\right]\cdot h_{\mathrm{quartz}}=1.181\cdot 10^{-4}\;m^{3}$. Hence, the total volume of argon is equal to $V_{\mathrm{ar},\mathrm{tot}}=V_{\mathrm{ar},\mathrm{ch}}+V_{\mathrm{ar},\mathrm{ext}}=2.00\cdot 10^{-4}\;m^{3}$. The mass of argon can be derived from the ideal gas equation as follows:

(2) $$m_{\mathrm{ar}}=\frac{pV_{\mathrm{ar},\mathrm{tot}}}{R_{\mathrm{specific},\mathrm{ar}}T_{\mathrm{start}}}=3.2123\times 10^{-4}\;\mathrm{kg};$$

where p=101,325 [Pa] is the atmospheric pressure, the initial gas temperature coincides with the room temperature $T_{\mathrm{start}}=303.15\;K$,$\;R_{\mathrm{specific},\mathrm{ar}}=208.1\;J/\left(\mathrm{kg}\cdot K\right)$ is the mass-specific gas constant, equal to the ratio between the ideal gas constant R [J/(mol·K)] and the gas molar mass M=0.039948 [kg/mol], see also [27,28]. Being the specific heat capacity at constant volume for argon $c_{v,\mathrm{ar}}=312.78\;J/\left(\mathrm{kg}\cdot K\right)$ (see e.g., [27]), the heat needed to warm its mass up to $T_{M}=660\;{}^{\circ}C$ amounts to $Q_{\mathrm{ar}}=m_{\mathrm{ar}}\cdot c_{v,\mathrm{ar}}\cdot \left(T_{M}-T_{\mathrm{start}}\right)=63.29\;J$.

Since the heating process of the inert gas occurs at constant volume, a pressure increase must be sustained by the quartz tube. In fact, during the transition from $T_{\mathrm{start}}=30\;{}^{\circ}C$ to $T_{M}=660\;{}^{\circ}C$, the pressure inside the chamber increases from $p_{\mathrm{start}}=101,325\;\mathrm{Pa}$ to $p_{\mathrm{end}}=p_{\mathrm{start}}\;T_{\mathrm{end}}/T_{\mathrm{start}}=311,897\;\mathrm{Pa}$. We can utilize the Barlow formula to evaluate the minimum tube thickness capable of sustaining such a pressure.

Knowing that the admissible tensile strength of the quartz glass equals $\sigma_{\mathrm{adm}}=47\;\mathrm{MPa}$, one has

(3) $$s_{\min}=\frac{\phi p_{\mathrm{end}}}{2\sigma_{\mathrm{adm}}}=0.3\;\mathrm{mm};$$

Therefore, a 2 mm thick quartz tube is by far suitable to the present purposes.

(iii) We calculate the heat needed to bring the sample holder (ref. 06, see Figure 7 and Figure 10) from room conditions at $T_{\mathrm{start}}$ to $T_{\mathrm{target}}>T_{M}$. Such a temperature guarantees that, by the conduction through the ceramic substrate, the melting temperature of the alloy $T_{M}$ is reached at the substrate/drop interface. We assumed $T_{\mathrm{target}}=1200\;{}^{\circ}C$, equal to the maximum operating temperature of $\mathrm{HASTELLOY}\;C22^{®}$, see Table A4. Being known the density and the specific heat capacity of this material, namely $\rho_{H.\;C22}=8690\;\mathrm{kg}/m^{3}$ and $c_{H.\;C22}=414\;J/\left(\mathrm{kg}\cdot {}^{\circ}C\right)$, the heat needed to warm its mass $m_{06}=0.28\;\mathrm{kg}$ amounts to $Q_{06\;\mathrm{heat}}=m_{06}\cdot c_{H.\;C22}\cdot \left(T_{\mathrm{target}}-T_{\mathrm{start}}\right)=136,535\;J$. This contribution is by far the most significant among those considered in this preliminary assessment. In conclusion, the minimal amount of heat $Q_{\mathrm{tot}}$ that must be generated at net of any loss amounts to $Q_{\mathrm{tot}}=Q_{\mathrm{drop}}+Q_{\mathrm{ar}}+Q_{06\;\mathrm{heat}}=137,039\;J$.

### 3.3. Thermal Resistance Network

**Figure 11:**
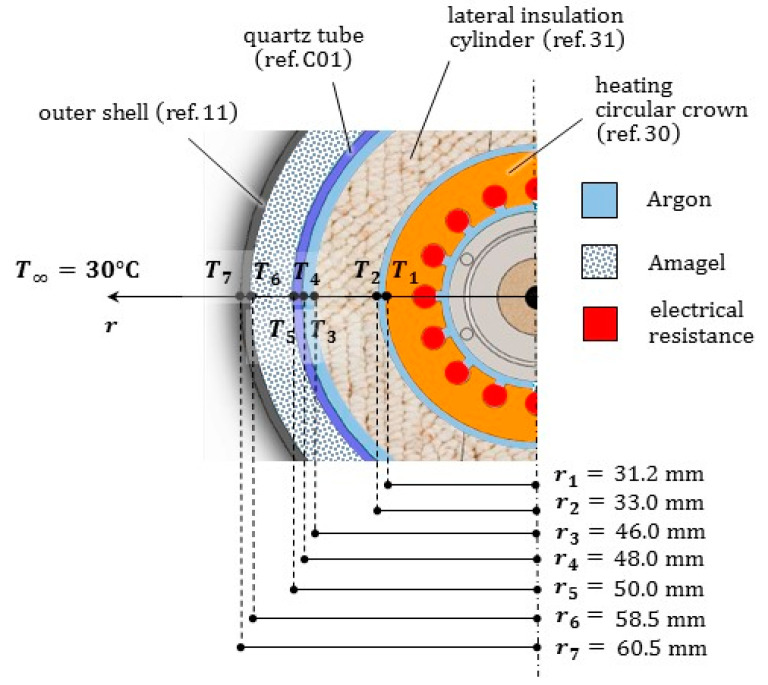
Top view of the coaxial material layers and of the gas filled cavities constituting the furnace, with the relevant size. Temperatures at each layer are indicated, to be computed by the thermal resistance network assuming a radial heat transfer mechanism.

We made recourse to the thermal resistance approach (see e.g., [26]) to assess the temperature profiles under steady conditions inside the furnace. Due to the peculiar geometry of the prototype (see Figure 11), we assumed a radial transfer mechanism of the heat from the ceramic circular crown (ref. 30), to the coaxial components ref. 31, ref. C01 and ref. 11 in the external layers, and to the sample holder (ref. 06) in the core of the furnace.

It is worth emphasizing that only the upper part of the ceramic circular crown includes an electrical resistance, corresponding to the height *L* of the sample holder: we will make reference to such a dimension for the next analytical developments. Moreover, the cavities between the sample holder (ref. 06) and the heating element (ref. 30), between the heating element and the insulation cylinder (ref. 31), and between the insulation cylinder and the quartz tube (ref. C01), exhibit small thicknesses (<2 mm), which become even smaller due to thermal expansions of the contiguous components. Therefore, gas argon in such cavities can be considered as quiescent, and the heat is transferred through them by conduction and radiation, acting in parallel. For the boundary condition over the outer face of the metal shell (ref. 11), convection and radiation were instead considered in parallel. An insulation composite panel of AMAGEL was jammed into the last cylindrical cavity between the quartz tube and the metal shell, 8.5 mm thick.

Moreover, we assumed that the heat from the sample holder (ref. 06), schematized by a unique computing point with the value $T_{\mathrm{target}}$, is transferred by conduction along the vertical direction to the ceramic disc, which in turn is in contact with the alloy droplet. The upper surface of the sample holder exchanges energy also in the form of radiation with the insulation walls surrounding the test chamber (assumed at $T_{M}$), and by free convection with the inert gas filling it (uniformly at $T_{M}$): all these contributions act in parallel.

**Figure 12:**
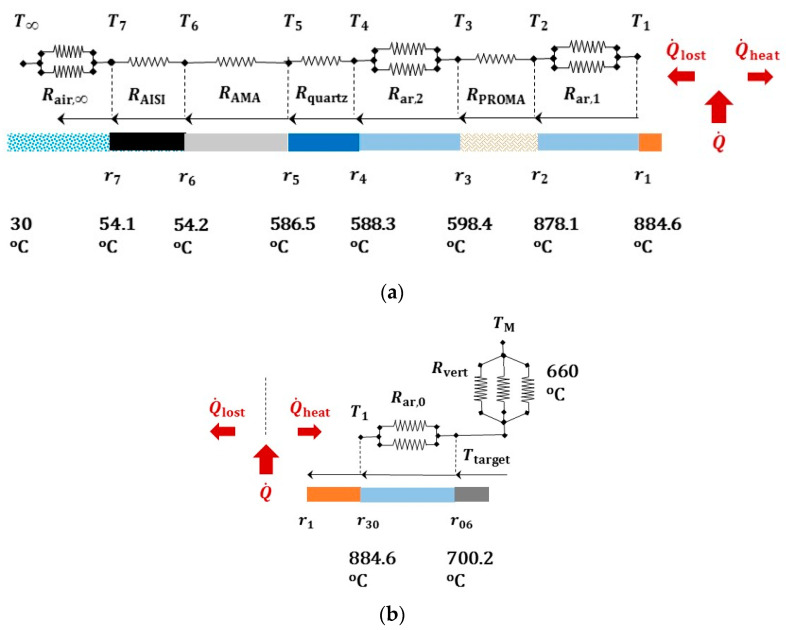
Steady state temperatures along the radius of the furnace (not in scale), predicted by the thermal resistance method for a generated power $\dot{Q}=60\;W$, and prescribed temperatures at the ends (*T*_∞_ of the environmental air and droplet melting temperature *T*_M_). With respect to the heating element at the temperature *T*_1_, scheme in (**a**) concerns the outer layers, that in (**b**) the inner ones. The subscripts ar and air denote argon and air properties, respectively.

Under the above simplifying assumptions, it is possible to draw a thermal resistance network aiming to predict the response of the multilayered prototype, see Figure 12. Through it we can estimate the temperatures profiles along the radius of the furnace, and assess which part of the heat rate $\dot{Q}$, generated by the ceramic element, gets “lost” ($\dot{Q}=\left|{\dot{Q}}_{\mathrm{heat}}\right|+\left|{\dot{Q}}_{\mathrm{lost}}\right|$). The power of the heater W, the melting temperature of the droplet $T_{M}$ and the temperature $T_{\infty }$ of the air in the outer environment must be prescribed.

Through the network of resistances, the steady state heat transfer problem is governed by the following system of three equations with three unknowns:

(4) $${\dot{Q}}_{\mathrm{lost}}=\frac{T_{1}-T_{\infty }}{R_{\mathrm{tot},\mathrm{ins}}};\;\;{\dot{Q}}_{\mathrm{heat}}=\frac{T_{M}-T_{1}}{R_{\mathrm{tot},\mathrm{heat}}};\;\;\dot{Q}={\dot{Q}}_{\mathrm{lost}}-{\dot{Q}}_{\mathrm{heat}};$$

where: ${\dot{Q}}_{\mathrm{lost}}>0$ since it flows toward the positive orientation of radial abscissa r, while ${\dot{Q}}_{\mathrm{heat}}<0$; $T_{1}$ denotes the temperature at the heating element; $R_{\mathrm{tot},\mathrm{insul}}$ is the resultant of the thermal resistances along the path of heat rate flow ${\dot{Q}}_{\mathrm{lost}}$, and analogously $R_{\mathrm{tot},\mathrm{heat}}\;$ for ${\dot{Q}}_{\mathrm{heat}}$.

In Table 2 and Table 3 symbol L=31 mm denotes the height of the sample holder, coincident with that of the heating part of the ceramic crown: all the calculations were referred to such a strip. The following values of the thermal conductivity k and of the convection coefficient h were considered for the specific conditions: $k_{\mathrm{ar}}=0.034\;W/\left(m\cdot {}^{\circ}C\right)$; $k_{\mathrm{proma}}=0.077\;W/\left(m\cdot {}^{\circ}C\right)$; $k_{\mathrm{quartz}}=1.5\;W/\left(m\cdot {}^{\circ}C\right)$; $k_{\mathrm{ama}}=0.019\;W/\left(m\cdot {}^{\circ}C\right)$; $k_{\mathrm{AISI}}=19.5\;W/\left(m\cdot {}^{\circ}C\right)$; $h_{\mathrm{ar}}=8.5\;W/\left(m^{2}\cdot {}^{\circ}C\right)$; $h_{\mathrm{air},\mathrm{in}}=8.7\;W/\left(m^{2}\cdot {}^{\circ}C\right)$ and $h_{\mathrm{air},\mathrm{out}}=40\;W/\left(m^{2}\cdot {}^{\circ}C\right)$.

In Table 2 the value of coefficient $h_{\mathrm{air},\mathrm{out}}$ corresponds to a condition of forced convection, with an air flow speed equal to 2 m/s (see Appendix B). Such an air flow can be generated by utilizing, for instance, small fans, with a 120 mm duct and a minimum air flux 200 cfm, blowing on the outer surface of the furnace.

In Table 3 $k_{\mathrm{cer}}=30\;W/\left(m\cdot {}^{\circ}C\right)$, $\phi_{\mathrm{cer}}=12$ mm, and $l_{\mathrm{cer}}=3\;\mathrm{mm}$ denote in the order the thermal conductivity, the diameter and the thickness of a ceramic disc of pure Alumina (${\mathrm{Al}}_{2}O_{3}$), herein considered as a reference substrate.

When prescribing a power $\dot{Q}=60\;W$, we found ${\dot{Q}}_{\mathrm{heat}}=-48\;W\;,\;{\dot{Q}}_{\mathrm{lost}}=+12\;W\;\mathrm{and}\;T_{1}=884\;{}^{\circ}C$. The resulting radial temperature profile is shown in Figure 12: it turns out to be compatible with the working temperatures of the selected materials. In particular, the insulation layers made of PROMAFORM^®^, 13 mm thick, and of AMAGEL^®^, 8.5 mm thick, with their high thermal resistance were apt to reduce temperatures of a few hundred degrees.

Of course, the above configuration was selected among alternative solutions. For instance, without the AMAGEL^®^ insulation panel, filling this cavity with air and considering in parallel convection (with coefficient $h_{\mathrm{air},\mathrm{in}}$) and radiation between the contiguous surfaces, the temperature at the outer wall raised to 72 °C; the heat power removed by forced convection over the considered strip amounted to 5 W. Instead, if exclusively natural convection were considered over the outer shell (with the coefficient $h_{\mathrm{air},\mathrm{in}})$, 100 °C and 150 °C were attained when including AMAGEL^®^ insulation layer or without it, respectively, corresponding in the order to 7 W and 15 W of removed heat power.

Table 4 outlines values of thermal resistances corresponding to the radiative heat transfer only, which were included in parallel in the thermal network with reference to gas filled cavities and to the outer boundary, see Figure 12. Details on the equations utilized for the radiative heat transfer were reported in the Appendix B, together with correlation formulae for the free and the forced convection coefficient *h*.

### 3.4. Finite Element Analyses

To simulate the steady state condition of the furnace, finite element analyses were performed under some simplifying assumptions in the Abaqus^®^/Standard platform utilizing implicit integration schemes, see [29]. Due to limited capabilities for heat transfer of the commercial code, these analyses included the conduction within the volume, the radiative exchange among inner surfaces forming enclosures or over the outer wall, whilst the convection was limited to the outer boundary. Therefore, the argon gas filling the test chamber and the other cavities was modelled as a further solid phase, with its own conductivity. Two axisymmetric models were developed: (i) one considering the same radial strip described by the network of thermal resistances; (ii) another one discretizing the longitudinal radial section of the entire furnace, with a rather accurate geometry.

**Figure 13:**
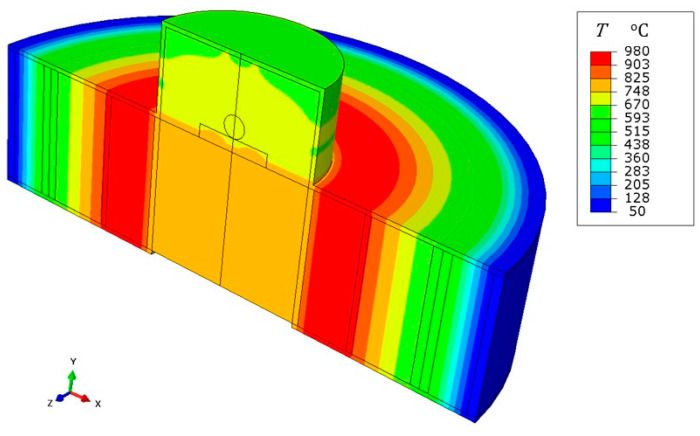
Steady state temperature field provided by an axisymmetric finite element model of the same radial strip utilized for the resistance network. Adiabatic conditions were considered at the top and at the bottom for the coaxial layers, whilst the drop melting temperature was prescribed over the test chamber walls. Three dimensional rendering was applied.

(i) Radial strip. The axisymmetric region with height *L* = 31 mm, extending from the core of the furnace to the outer wall (see Figure 11 and Figure 12), was discretizated by means of about 2500 four node elements, referred to as DCAX4 in the Abaqus^®^ element library. The heat power generated by the ceramic circular crown was set equal to that utilized for the network exercise, i.e., $\dot{Q}=60W$, although expressed as a bulk thermal load. The melting temperature of the droplet *T*_M_ = 660 °C was prescribed over the walls of the test chamber. Moreover, to enforce a radial heat transfer process, the coaxial layers within the strip were enclosed by adiabatic walls above and below (with $\dot{Q}=0$). As shown in Figure 13, this model provided a radial temperature profile in agreement with the network of thermal resistances.

(ii) Longitudinal radial section of the furnace. A rather accurate geometry of the furnace was discretized by means of 17,500 triangular finite elements, referred to as DCAX3 in the Abaqus^®^ element library. To attain the molten temperature of the droplet, we increased significantly the heat power of the ceramic element; this circumstance is not surprising, since herein the heat can be transferred also along the vertical direction. The thermal parameters governing conduction, radiation and convection over the outer boundary were not modified with respect to the network of resistance.

**Figure 14:**
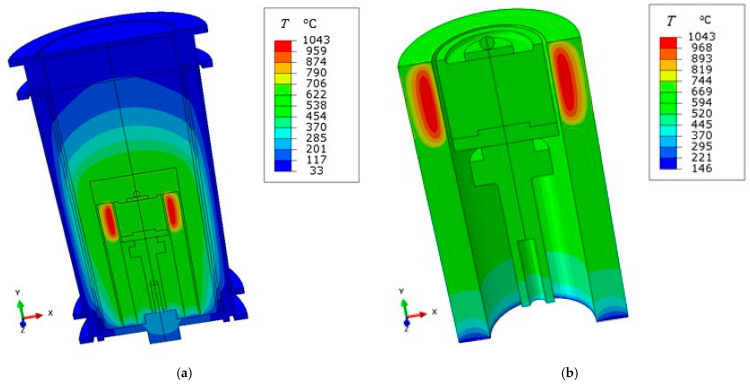
(**a**) Steady state temperature field provided by an accurate axisymmetric finite element model of the entire furnace. (**b**) Detail with a different temperature and color interval, including only the sample holder with its rotation transmission system, and the heating cylindrical element. Three dimensional rendering was applied.

As shown in Figure 14a, this second model revealed an important gradient of temperatures along the height of the furnace: it indicated that temperatures remain low over the outer wall, in agreement with the prediction of the approximated network, and that an effective insulation was operated by the thick roof of the test chamber. Over the lower header, instead, close to the feedthrough, this model predicted a local spike of temperatures approaching 130 °C, see Figure 14b. This circumstance, if confirmed, would suggest us to insert an additional insulation layer between the base header and the anchorage system. It is worth recalling that we could not include in the above finite element analyses computational fluid dynamics (CFD) simulations of the convective heat transfer inside the gas filled cavities, and that the conduction process is less effective in removing and transferring heat with respect to convection. From this point of view, the present results could be affected by a spurious amplification of the temperature gradients, which ultimately require an accurate validation step.

## 4. Discussion

Several equipment for high temperature in situ testing under X-ray microtomography have been designed for use in a synchrotron beamline: a part of them were also capable of applying mechanical loading on “hot” samples. However, to our knowledge, none of such devices was explicitly conceived for carrying out in situ sessile droplet tests, namely, for wettability experiments at high temperatures and under inert atmosphere on a molten droplet/solid substrate pair. To assemble a furnace prototype suitable for the purpose, the authors faced courageously several problems with a novel design, optimizing each component and material. The present study opens perspectives of special interest to the researchers who have at disposal only commercial X-ray systems with low power tubes, exhibiting severe constraints on the geometry and the weight of the samples, and with the need to install/remove easily the auxiliary equipment without modifying or damaging the preexisting setup.

The basic design choice, that affected the entire project, concerned which parts of the furnace should rotate, and which ones instead should stand still during the acquisition of the angular projections. The solution adopted allows the sample holder alone to rotate fastened to the rotary stage of the tomograph by means of a transmission system, playing at the same time an insulation and a sealing role. The remaining degrees of freedom of the sample holder are in common with those of the furnace, which is stably anchored to the translatory stage of the tomograph. This choice enables the user to position accurately the entire furnace, and to align the sample with the X-ray beam, properly tuning the magnification. In particular, the heating element of the furnace remains still during the acquisition step. Its cables and wires, suitably coated for the high temperatures, do not twist; they pass through small holes in the lower header and are connected to the PID controller and to the power supply.

For the wettability experiments at high temperatures, an inert atmosphere or a high vacuum is required, to prevent the molten alloy droplet from the oxidation: the air can be admitted exceptionally when testing pure silver or gold. Due to the presence of the quartz tube sealed by O-rings, and of the furnace headers including pipes for inlet and outlet gas flow, the test chamber can be filled with an inert gas such as argon. A slight overpressure must be guaranteed, to avoid contamination of the inner atmosphere with oxygen atoms. At the same time, high purity fused quartz glass exhibits a low X-ray attenuation, as confirmed by preliminary transmission tests carried out on solid droplets at room temperature.

**Figure 15:**
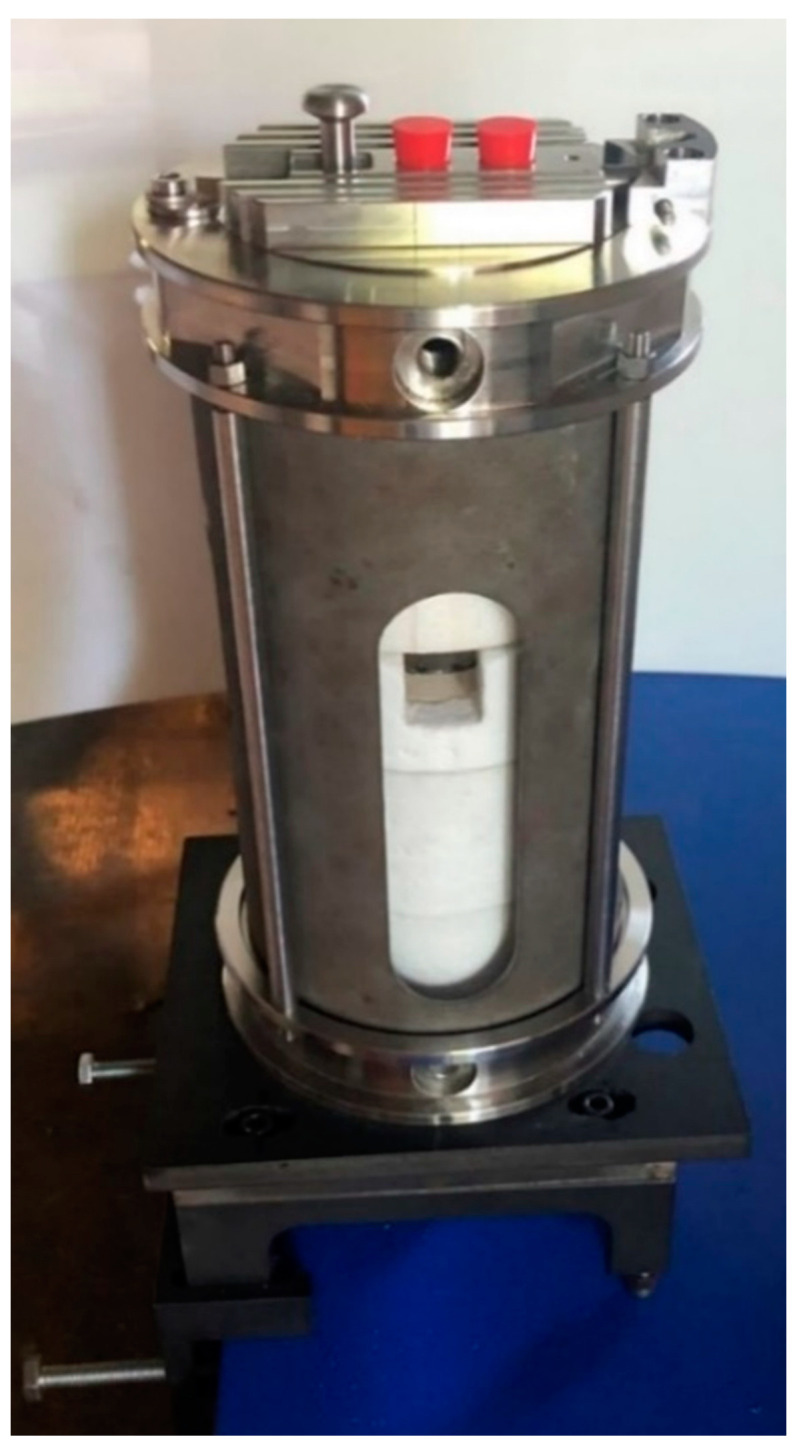
Picture of the furnace prototype after assembling.

In the presence of uncertain values for several material properties over a wide temperature range, the analytical calculations and the finite element simulations of heat transfer outlined above represented a compromise between accuracy and simplicity, and constituted a valid support for design. Although the considered approaches, resting on diverse simplifying assumptions, exhibited serious limitations, a satisfactory overall agreement of the results was met: the steady state temperatures turned out to be compatible with the selected materials, and the insulation layers played an important role despite the present constraints on the geometry and on the X-ray attenuation. The thermal resistance network indicated the need of cooling the outer wall of the furnace by forced convection: to this purpose small fans can be used, positioned inside the cabinet. The finite element analyses of the furnace confirmed the effectiveness of the insulation at the outer wall and over the roof. Instead, it revealed a spike of temperatures closely to the feedthrough at the base header, which might require a further insulation layer. Now that we successfully assembled the furnace prototype, see Figure 15, we can confirm through experimental measurements the accuracy of the above thermal predictions. In particular, we intend to correlate through a robust nonlinear regression the measurement of the thermocouple in the ceramic heater, providing the feedback signal for the PID controller, with the temperatures at the sample location, utilizing at rest high temperature thermometers and eutectic crucibles, see e.g., [30].

As an alternative to fans, a water cooling system can be utilized; however, also in this case the diverse components (pump/reservoir, hoses, radiator) must be carefully selected to fulfill the geometry constraints and to permit an optimal configuration. The ducts for the coolant circulation could be realized within the outer cylindrical shell of the furnace. It is worth noting that the cabinet is equipped with a compact lead door opening with hinges, located behind the X-ray tube for maintenance and inspection and fastened by screws during the normal activities. The authors designed another door, satisfying all the radiation protection requirements, with an identical geometry except the presence of a wide shielded passage for the cables/wires, which follows a not straight path (with sharp bends). At the same time, such a passage can guarantee some air exchange with the outer room and hence a light air conditioning. Especially for the in situ experiments, in fact, possibly lasting several hours, the thermal drift of the focal spot has to be prevented; it has been identified as one of the major causes of the positional instability of the angular projections.

The testing step for the novel furnace is expected to be complex, well beyond the thermal behavior, and will require a long effort. For instance, stability of the molten droplet during the rotation must be ensured, since even small oscillations can generate artifacts in the tomographic reconstruction; roller bearing rotary stage may be not sufficient to prevent vibrations and wobbling, and an air bearing equipment might be necessary. Image quality must be carefully optimized, at varying the brazing alloy/substrate pair, the magnification and the beam parameters, ensuring that the droplet profile is correctly retrieved. To this purpose, common edge detectors for digital image processing can be utilized [31]. Finally, wettability measurements provided by the present approach, in terms of contact angle and surface tension, must be critically validated against literature data. Clearly, once completed the entire validation step for the selected reference materials at 660 °C, our aim is to perform wettability tests on a wide range of alloy/substrate pairs at both lower and higher temperatures.

At lower temperatures, the soldering joints, used for the electronic circuits, are of extreme interest for the industry. Due to the inherent toxicity of Pb, the environmental regulations worldwide prompted the replacement of all the Lead-Containing solder alloys, in particular the Sn-Pb alloy, with suitable Lead-Free Solders, such as for instance SnCu (*T*_sol_ = 224.6 °C, *T*_M_ = 227 °C) or SnBi (*T*_sol_ = 134.7 °C, *T*_M_ = 138 °C), being *T*_sol_ the characteristic temperature of solidification and *T*_M_ the melting temperature, see e.g., [32]. These alloys are valid candidates to replace Pb-containing solders, since their solidus temperatures are high enough to maintain joint reliability during thermomechanical fatigue.

At higher temperatures, the “reactive wetting” deserves further investigation; this phenomenon occurs when the substrate reacts with the molten alloy droplet, giving rise to new compounds. For instance, it is the case of the alloy CuAg–3.8at%TiSi on a SiC substrate, at 880 °C [33]. In that scenario X-ray microtomography can allow one to reconstruct the possibly curved interface between the droplet and the substrate, and to assess the actual penetration of the braze into the ceramic substrate. Since one of the factors limiting the furnace heating was indeed the insulation, in the case of higher temperatures the recourse to a water cooling system might be preferable.

It is worth considering that, once the furnace has cooled down, a solidified droplet joined to the substrate becomes available. Non conventional mechanical experiments, referred to as push off tests (see e.g., [34]), can be carried out also on such a system, to assess the average shear strength of the joint and its fracture energy, for instance by means of full field kinematic data and inverse analyses [35,36,37]. X-ray imaging can still be utilized to monitor the experiment at room temperature, if an in situ loading apparatus is available. This further investigation on a solid assembly, carried out after the sessile drop test, would permit to correlate for the same sample thermodynamic quantities at high temperatures, widely utilized in materials science, with other quantities more typical of mechanical engineering.

The rapid growth of in situ tests monitored by X-ray tomography, documented also by this study, is due to the convergence of several favorable factors, such as the rising industrial demand for high performing materials, the enhanced capabilities for the microstructure control and, not last, the availability on the market of less expensive X-ray systems. Such in situ methodologies are favoring the research and the innovation concerning advanced materials but also the progress of the X-ray technology itself. The short-term objective is to realize synergies among experimental, theoretical and computational approaches for the material characterization and design.

## Figures and Tables

**Table 1:** Some X-ray linear attenuation coefficients at 50 keV energy, from [25].

Material	X-ray Linear Attenuation Coefficient µ [cm^−1^]
Fused quartz glass	0.684
Air (dry)	0.000254
Ar (Argon)	0.00109
Al_2_O_3_ (Alumina)	1.14
Ag (Silver)	92.1
Cu (Copper)	21.61

**Table 2:** Main thermal resistances corresponding to the coaxial material layers and to the gas filled cavities outside the heating element. Only the contributions of conduction and convection heat transfer were shown, specified by the subscripts cond and conv, respectively; ar and air denote argon and air.

Thermal Resistances of the Outer Layers for Conduction/Convection			
$R_{\mathrm{cond}\;\mathrm{ar},1}$	$=\frac{\ln\left(r_{2}/r_{1}\right)}{2\pi L\cdot k_{\mathrm{ar}\;}}=$	8.4696	°C/W
$R_{\mathrm{cond}\;\mathrm{proma}}$	$=\frac{\ln\left(r_{3}/r_{2}\right)}{2\pi L\cdot k_{\mathrm{proma}}}=$	22.290	°C/W
$R_{\mathrm{cond}\;\mathrm{ar},2}$	$=\frac{\ln\left(r_{4}/r_{3}\right)}{2\pi L\cdot k_{\mathrm{ar}}}=$	6.4265	°C/W
$R_{\mathrm{cond}\;\mathrm{quartz}}$	$=\frac{\ln\left(r_{5}/r_{4}\right)}{2\pi L\cdot k_{\mathrm{quartz}}}=$	0.1435	°C/W
$R_{\mathrm{cond}\;\mathrm{AMA}}$	$=\frac{\ln\left(r_{6}/r_{5}\right)}{2\pi L\cdot k_{\mathrm{AMA}}}=$	42.420	°C/W
$R_{\mathrm{cond}\;\mathrm{AISI}\;}$	$=\frac{\ln\left(r_{7}/r_{6}\right)}{2\pi L\cdot k_{\mathrm{AISI}}}=$	0.0089	°C/W
$R_{\mathrm{conv}\;\mathrm{air},\infty }$	$=\frac{1}{2\pi Lr_{7}\cdot h_{\mathrm{air},\mathrm{out}}}=$	2.1215	°C/W

**Table 3:** Thermal resistances for the elements internal to the heating cylinder. Only the contributions of conduction and convection heat transfer were shown, specified by the subscripts cond and conv. Convection resistance refers to the upper face of the sample holder.

Thermal Resistances of the Inner Layers for Conduction/Convection			
$R_{\mathrm{cond}\;\mathrm{ar},0}$	$=\frac{\ln\left(r_{30}/r_{06}\right)}{2\pi L\cdot k_{\mathrm{ar}}}=$	11.161	°C/W
$R_{\mathrm{cond},\mathrm{sub}.\mathrm{cer}.}$	$=\frac{l_{\mathrm{cer}}}{A_{\mathrm{cer}}\cdot k_{\mathrm{sub}\;\mathrm{cer}}}=$	0.8842	°C/W
$R_{\mathrm{conv},\mathrm{sample}}$	$=\frac{1}{A_{06}\cdot h_{\mathrm{ar}}}=$	112.44	°C/W

**Table 4:** Thermal resistances corresponding to radiative heat transfer. Subscripts ar and air denote the vertical cavities filled by argon or air, respectively; sample indicates the upper surface of the sample holder.

Thermal Resistances for Radiative Heat Transfer		
$R_{\mathrm{rad}\;\mathrm{sample}\;}=$	24.3	°C/W
$R_{\mathrm{rad}\;\mathrm{ar},0}=$	5.14	°C/W
$R_{\mathrm{rad}\;\mathrm{ar},1}=$	0.55	°C/W
$R_{\mathrm{rad}\;\mathrm{ar},2}=$	0.92	°C/W
$R_{\mathrm{rad}\;\mathrm{air},\infty }=$	19.9	°C/W

## Data Availability

Data available within this article.

## Appendix A. List of Main Furnace Components

**Table A1:** List of the main components of the furnace prototype, divided into three groups: the furnace, the upper header and the base structure. A reference code allows the reader to identify each component when indicated within the text or in the figures.

Reference Code	Material	Component Description
04	AISI 304	Furnace lower header.
06	HASTELLOY	Sample holder.
08	ORVICAL·1500	Mushroom-shaped insulating rotary axis.
09	ORVICAL·1500	Insulating pad.
11	AISI 304	Outer cylindrical shell.
19	AISI 304	Pillars.
30	42TE	Heating cylindrical element.
31	PROMAFORM	Insulation cylinder.
32	PROMAFORM	Insulation roof of the testing chamber.
C01	QUARTZ GLASS	Quartz glass tube.
C06	STAINLESS STEEL	Rotary Motion Feedthrough.
C07	FPA	Sealing O-rings.
C10	AISI 304	Pipe connections for inert gas.
03	AISI 304	Furnace upper header.
13	AISI 304	Finned lid plate.
20	AISI 304	Connection between the feedthrough shaft and the rotary stage.
21	CARBON STEEL	C-shaped clamp.
24	CARBON STEEL	Trilithon structure for the furnace base.

## Appendix B. Equations for Radiative and Convective Heat Transfer and Relevant Parameters

The contribution of radiative heat transfer (see e.g., [26,38]) can be included in the network of thermal resistances analogously to what is carried out for conduction and convection, although relevant resistance explicitly depends on the unknown temperatures.

The upper surface of the sample holder (ref. 06), assumed opaque, gray and diffuse, exchanges energy in the form of radiation with the insulation walls of the test chamber, which completely surround it and exceed by far its area. The net rate of radiation for the sample holder upper face and its thermal resistance are then provided by the following formulae (see [26]):

(A1) $${\dot{Q}}_{\mathrm{rad}}=\varepsilon_{06}\sigma A_{06}\left(T_{06}^{4}-T_{\mathrm{surr}}^{4}\right);\;\;R_{\mathrm{rad}}={\left\{\varepsilon_{06}\sigma A_{06}\left(T_{06}^{2}+T_{\mathrm{surr}}^{2}\right)\left(T_{06}+T_{\mathrm{surr}}\right)\right\}}^{-1}$$

being σ = 5.6704 × 10^−8^ [Wm^−2^ K^−4^] the Stefan-Boltzmann constant, ε_06_ and A_06_ the emissivity and the area of the face.

For the radiative heat exchange between the opaque, gray and diffuse isothermal vertical cylindrical surfaces of two contiguous layers inside the furnace, say 1 and 2, we utilized the formula (see e.g., [38])

(A2) $${\dot{Q}}_{1\to 2}=\frac{\sigma \left(T_{1}^{4}-T_{2}^{4}\right)}{\frac{1-\varepsilon_{1}}{\varepsilon_{1}A_{1}}+\frac{1}{A_{1}F_{1\to 2}}+\frac{1-\varepsilon_{2}}{\varepsilon_{2}A_{2}}};\;R_{\mathrm{rad}}={\left\{\frac{\left(T_{06}^{2}+T_{\mathrm{surr}}^{2}\right)\left(T_{06}+T_{\mathrm{surr}}\right)}{\frac{1-\varepsilon_{1}}{\varepsilon_{1}A_{1}}+\frac{1}{A_{1}F_{1\to 2}}+\frac{1-\varepsilon_{2}}{\varepsilon_{2}A_{2}}}\right\}}^{-1}$$

where: symbol $F_{1\to 2}$ indicates the view factor (or configuration factor) from surface 1 to surface 2, i.e., the percentage of radiative energy leaving 1 and impinging on 2; *ε*_1_ and *A*_1_ denote emissivity and area of surface 1, and analogously for surface 2. It is worth noting that the above equation is strictly valid only when the two surfaces above constitute an enclosure, as it occurs when the coaxial cylinders are infinitely long. Its application to the present context represents a simplifying approximation, since we are neglecting the circular crowns at the top and at the bottom of the small reference subvolumes, and the relevant interactions. When the coaxial cylinders possess a finite length *L*, say r_1_ and r_2_ the inner (convex) and the outer (concave) surface radii, respectively, one has: $0\leq F_{2\to 1}<1$: value of $F_{2\to 1}$ can be parametrized with respect to the ratios *L/*r_2_ and r_1_/r_2._. Moreover, the following relationships among view factors hold: $A_{1}F_{1\to 2}=A_{2}F_{2\to 1}$ (reciprocity, between any two surfaces); $\underset{j}{\sum }F_{i\to j}=1$ (summation of view factors extended to all surfaces *j* forming an enclosure). For the inner convex surface we have $F_{1\to 1}=0$ (no self-radiation), whilst for the concave one, $F_{2\to 2}>0$.

In the heat transfer calculations developed above, we assumed the following values of emissivity: HASTELLOY, ε=0.2; 42TE ceramics, ε=0.9; PROMAFORM ε=0.9; quartz ε=0.55; AISI 304, ε=0.6. For further details, the reader is forwarded to [38,39,40,41]. By setting equal all the emissivities to a unit value, as the surfaces were ideal black bodies unable of reflecting any radiation, by the network of resistance one would obtain a temperature at the heater lower of about one hundred degrees, whilst the outer wall temperature would remain unchanged. This circumstance is not too surprising, since emissivity selected for the HASTELLOY sample holder was rather low. For the ideal scenario with $\varepsilon_{i}\to 1$, Equation (A2) returns a correct value.

For the estimation of the natural convection coefficient *h* at a vertical surface, the following correlation equation was utilized (see e.g., [26])

(A3) $$\mathrm{Nu}=\frac{hL}{k}={\left\{0.825+\frac{0.387\;{\mathrm{Ra}}^{1/6}}{{\left[1+{\left(0.492/\mathrm{Pr}\right)}^{9/16}\right]}^{8/27}}\right\}}^{2}$$

where *L* denote a characteristic length parameter, *k* the thermal conductivity of the fluid, symbols Nu, Ra, Pr indicate the nondimensional Nusselt, Rayleigh and Prandtl numbers (in the jargon referred to as groups), respectively. Rayleigh number is usually expressed as the product Ra = (Pr Gr), being Gr the Grashof number. Grashof and Prandtl nondimensional groups for a fluid are defined as follows

(A4) $$\mathrm{Gr}=\frac{g\rho^{2}L^{3}\beta \left(T_{s}-T_{\infty }\right)}{\mu^{2}};\;\mathrm{Pr}=\frac{\mu c_{P}}{k};$$

being g the gravity acceleration, *ρ* and *β* the fluid density and its thermal expansion coefficient, *T*_s_ and *T*_∞_ the surface and the environmental temperature, *μ* the dynamic viscosity, *c*_P_ the fluid specific heat capacity at constant pressure.

In conditions of forced convection over an isothermal flat plate, with an air flow having velocity *V**_∞_*, recourse is made to the following correlation formula providing the average Nusselt number

(A5) Nu=hLk=0.664 Re0.5Pr0.33 

being the Reynolds number defined as

(A6) Re=ρ V∞Lμ

## Appendix C. Tables of Material Properties

**Table A2:** Mechanical and thermal properties of Quartz Glass provided by Squall International bv (NL), see http://squallquartz.com/ (Accessed Date: 9 November 2021).

PROPERTIES	Quartz Glass
Density	$2200\;\mathrm{kg}/m^{3}$
Compressive strength	1150 MPa
Tensile strength	50 MPa
Modulus of elasticity	7000 MPa
Poisson ratio	0.17
Max Operating Temperature	1100 °C
Peak Temperature	1300 °C
Thermal Expansion coeff.	$5.1\cdot 10^{-7}{\;K}^{-1}$
Thermal Conductivity	1.46 W/(m·°C)

**Table A3:** Mechanical and thermal properties of AISI 304. See https://www.makeitfrom.com/material-properties/AISI-304-S30400-Stainless-Steel (Accessed Date: 9 November 2021).

PROPERTIES		AISI 304
Density		$7930\;\mathrm{kg}/m^{3}$
Tensile strength		515 MPa
Thermal Expansion coeff.at	100 °C	$1.68\cdot 10^{-5}{\;K}^{-1}$
	400 °C	$1.78\cdot 10^{-5}{\;K}^{-1}$
	600 °C	$1.88\cdot 10^{-5}{\;K}^{-1}$
	800 °C	$2.02\cdot 10^{-5}{\;K}^{-1}$
Thermal Conductivity at	20 °C	15.00 W/(m·°C)
	100 °C	16.30 W/(m·°C)
	200 °C	17.50 W/(m·°C)
	400 °C	19.90 W/(m·°C)
	500 °C	21.50 W/(m·°C)
	600 °C	22.50 W/(m·°C)
	800 °C	25.10 W/(m·°C)

**Table A4:** Mechanical and thermal properties of $\mathrm{HASTELLOY}\;C22^{®}$. See https://fondinox.it/materiale/hastelloy-c276/ (Accessed Date: 9 November 2021).

PROPERTIES		$\mathrm{HASTELLOY}\;C22^{®}$
Density		$8690\;\mathrm{kg}/m^{3}$
Tensile strength		690 MPa
Elastic Modulus		206 GPa
Melting Temperature		1399 °C
Specific Heat Capacity		414 J/(m·°C)
Thermal Expansion coeff.		15.5 μm/(m·°C)
Thermal Conductivity at	100 °C	11.10 W/(m·°C)
	200 °C	13.40 W/(m·°C)
	300 °C	15.50 W/(m·°C)
	400 °C	17.50 W/(m·°C)
	500 °C	19.50 W/(m·°C)
	600 °C	21.30 W/(m·°C)

**Table A5:** Mechanical and thermal properties of the three insulating materials, candidates for components (ref. 08) and (ref. 09). See https://www.corning.com/worldwide/en/products/advanced-optics/product-materials/specialty-glass-and-glass-ceramics/glass-ceramics/macor.html (Accessed Date: 9 November 2021); http://www.goodfellow.com/I/Duratec-750-Ceramica-Lavorabile-Barra.html (Accessed Date: 9 November 2021); https://www.orvim.it/eng/composite-materials/ (Accessed Date: 9 November 2021).

PROPERTIES		$\mathrm{MACOR}\;750^{®}$	$\mathrm{DURATEC}\;750^{®}$	ORVICAL 1500
Density		$2520\;\mathrm{kg}/m^{3}$	$1400\;\mathrm{kg}/m^{3}$	$1050\;\mathrm{kg}/m^{3}$
Compressive strength		345 MPa	55 MPa	22 MPa
Flexural strength		−	23 MPa	11 MPa
$\mathrm{Thermal}\;\mathrm{Expansion}\;\mathrm{coeff}.\;\left(\mathrm{at}\;T_{\mathrm{cl}}\right)$		$1.26\cdot 10^{-5}\;{}^{\circ}C^{-1}$	$6.60\cdot 10^{-6}\;{}^{\circ}C^{-1}$	0.1÷0.65%
$\mathrm{Peak}\;\mathrm{Temperature}\;\left(T_{p}\right)$		1000 °C	−	1600 °C
$\mathrm{Classification}\;\mathrm{temperature}\;\left(T_{\mathrm{cl}}\right)$		800 °C	1000 °C	1500 °C
Thermal Conductivity at	400 °C	1.46 W/(m·°C)	0.49 W/(m·°C)	0.25 W/(m·°C)
	600 °C	1.46 W/(m·°C)	0.49 W/(m·°C)	0.27 W/(m·°C)
	800 °C	1.46 W/(m·°C)	0.49 W/(m·°C)	0.29 W/(m·°C)

**Table A6:** Mechanical and thermal properties of ${\mathrm{PROMAFORM}}^{®}\;\mathrm{ECO}\;1250$, see https://www.promat-hpi.com/it-it/products/promaform-1600 (Accessed Date: 9 November 2021).

PROPERTIES		${\mathrm{PROMAFORM}}^{®}\;\mathrm{ECO}\;1250$
Density		$320\;\mathrm{kg}/m^{3}$
Compressive strength		0.25 MPa
$\mathrm{Classification}\;\mathrm{temperature}\;\left(T_{\mathrm{cl}}\right)$		1250 °C
Thermal Conductivity at	200 °C	0.06 W/(m·°C)
	400 °C	0.08 W/(m·°C)
	600 °C	0.11 W/(m·°C)
	800 °C	0.12 W/(m·°C)
	1000 °C	0.16 W/(m·°C)

**Table A7:** Properties of ${\mathrm{AMAGEL}}^{®}\;A2\;650$, see https://amagel.it (Accessed Date: 9 November 2021).

PROPERTIES		${\mathrm{AMAGEL}}^{®}\;A2\;650$
Density		$200\;\mathrm{kg}/m^{3}$
Working temperature		−50/+650 °C
Thermal Conductivity at	0 °C	0.015 W/(m·°C)
	100 °C	0.020 W/(m·°C)
	350 °C	0.025 W/(m·°C)
	460 °C	0.030 W/(m·°C)
	650 °C	0.035 W/(m·°C)
